# Parents' information and support needs when their child is diagnosed with type 1 diabetes: a qualitative study

**DOI:** 10.1111/hex.12244

**Published:** 2014-07-29

**Authors:** David Rankin, Jeni Harden, Norman Waugh, Kathryn Noyes, Katharine D. Barnard, Julia Lawton

**Affiliations:** ^1^ Centre for Population Health SciencesUniversity of EdinburghEdinburghUK; ^2^ Warwick Medical SchoolUniversity of WarwickCoventryUK; ^3^ Royal Hospital for Sick ChildrenEdinburghUK; ^4^ Human Development and HealthUniversity of SouthamptonSouthamptonUK

**Keywords:** parents' experiences, pediatric diabetes, qualitative research, type 1 diabetes

## Abstract

**Aim and objective:**

The aim of this study was to describe and explore parents' information and support needs when their child is diagnosed with type 1 diabetes, including their views about the timing and chronology of current support provision. Our objective was to identify ways in which parents could be better supported in the future.

**Design and participants:**

Semi‐structured interviews were conducted with 54 parents of children with type 1 diabetes in four paediatric diabetes clinics in Scotland. Data were analysed using an inductive, thematic approach.

**Findings:**

Parents described needing more reassurance after their child was diagnosed before being given complex information about diabetes management, so they would be better placed psychologically and emotionally to absorb this information. Parents also highlighted a need for more emotional and practical support from health professionals when they first began to implement diabetes regimens at home, tailored to their personal and domestic circumstances. However, some felt unable to ask for help or believed that health professionals were unable to offer empathetic support. Whilst some parents highlighted a need for support delivered by peer parents, others who had received peer support conveyed ambivalent views about the input and advice they had received.

**Conclusions:**

Our findings suggest that professionals should consider the timing and chronology of support provision to ensure that parents' emotional and informational needs are addressed when their child is diagnosed and that practical advice and further emotional support are provided thereafter, which takes account of their day‐to‐day experiences of caring for their child.

## Background

Type 1 diabetes (T1D) is one of the most common chronic conditions in childhood. The incidence has been rising by 3–4% per year in many countries, with the largest age‐specific rise occurring amongst children aged under 5 years.^1^, ^2^ In the UK, there are approximately 26,500 children living with T1D^3^, ^4^ and the incidence (24.5–26/100 000 children per year) is amongst the highest in the world.^2^, ^5^, ^6^ Most newly diagnosed children are now treated with one or two injections of long‐acting basal insulin and injections of short‐acting insulin at meal times. This regimen requires parents, who assume most responsibility for day‐to‐day management of pre‐adolescent young children, to undertake frequent (at least four per day) checking of blood glucose levels, determine and administer insulin doses, count carbohydrates, be aware of physical activity levels and prevent hypo‐ and hyperglycaemia.^7^, ^8^

These responsibilities place considerable emotional demands on parents, particularly mothers,^9^ who normally assume most responsibility for their child's diabetes care. As several qualitative studies have highlighted, parents whose children have been recently diagnosed with T1D describe feeling shocked, exhausted and out of control.^10^, ^11^, ^12^, ^13^ Such parents also report feeling anxious about administering injections, being frightened about episodes of hypoglycaemia and experiencing difficulties developing new and making adjustments to existing family routines, such as shopping, cooking and outings.^10^, ^11^, ^12^, ^13^, ^14^, ^15^, ^16^, ^17^ Parents of newly diagnosed children have also been shown to experience higher levels of clinically significant anxiety and stress than parents of healthy children and a higher prevalence of clinically significant depressive symptoms.^18^, ^19^, ^20^, ^21^, ^22^ Furthermore, parents have reported feelings of frustration, guilt and anger due to the difficulties of adhering to complex regimens and feelings of personal failure when regimens are not strictly followed.^23^

Whilst the emotional, psychological and practical impact of a child's diagnosis on parents has been comprehensively reported,^10^, ^14^, ^20^ there has been relatively little exploration of parents' views about the information and support they receive in the aftermath of diagnosis in order to undertake their new caregiver responsibilities. There has also been no research which has looked at parents' views about the timing and chronology of current support provision, despite this being shown to be a salient issue for adults newly diagnosed with diabetes.^24^, ^25^ Furthermore, when parents' post‐diagnostic accounts of support have been reported, the findings have often focused on those with very young children (typically, 4 years and under),^12^, ^26^, ^27^ have tended to be brief, and have been presented as part of their broader experiences of making adaptations to family life, acquiring skills and learning how to manage their child's T1D over time.^10^, ^12^, ^14^, ^15^, ^16^ In this paper, we report findings which emerged from a qualitative study originally established to explore the experiences of parents who care for children aged under 12 years, diagnosed with T1D. Early into our interviews it became apparent that all parents wanted and expected to discuss their experiences of diagnosis in‐depth together with their views about how they could have been better supported. In response to this emerging finding, we adapted our topic guide to allow for a detailed exploration of parents' experiences of, views about, and need for, information and support after their child's diagnosis. In doing so, our objective was to inform recommendations for how parents could be better supported in order to help alleviate their anxiety and distress and to foster effective diabetes management when they first begin to care for a child with T1D.

## Methods

### Research design

In‐depth semi‐structured interviews were undertaken with parents of children aged 12 years and under who had been diagnosed with T1D. In‐depth interviews were used as these afforded the flexibility needed for parents to share their own understandings and experiences and to raise and discuss issues which were salient to them, including those not anticipated at the study's outset.^28^ The study used an emergent design, in which data collection and analysis took place simultaneously, informed by the principles of grounded theory research.^29^ This enabled the issues identified in the early interviews to inform the areas explored in later ones.

### Sample and recruitment

Fifty four parents (mothers and fathers) of children aged 2–12 years were recruited using an opt‐in method by health professionals at the time of their child's consultation. Parents who attended consultations were recruited from paediatric diabetes clinics located in four health boards which serve diverse (urban, rural and remote rural) catchment areas across Scotland. Purposive sampling was used to ensure diversity of location, occupational status (full‐time/part‐time), relationship status and their child's gender and demographic/disease characteristics (Table 1). During recruitment, parents who attended a consultation alone were asked to enquire whether their partner would also like to participate in an interview. If two parents were present during a consultation, both were offered the opportunity to take part. A recruitment log was maintained and reviewed on a weekly basis by members of the research team during the study's recruitment period. These weekly reviews were used to inform discussions with recruiting staff to ensure that parents in all of the groupings in Table 1 were included in the final sample. To assist this process, recruiting staff had access to clinical records to inform participant selection. All participants who opted‐in completed a written consent form prior to participation. Permission was also sought to re‐approach parents, if necessary, by telephone to clarify information or explore emergent issues during data collection/analysis. Recruitment continued until no new findings or themes were identified in new data collected.

**Table 1:** Demographic characteristics of interview participants and their children

Characteristic	*N*	%	Mean ± SD and range
Parents (*n* = 54)^a^			
Female (mothers)^b^	38	70.4	
Age – all parents (years)			40.6 ± 6.1, range 25–51
Mothers Age (years)			40.0 ± 5.6, range 25–51
Fathers Age (years)			42.1 ± 7.0, range 27–51
Biological parents living together (data from 40 interviews)	28	70.0	
Current employment status			
Full‐time	19	35.2	
Part‐time	18	33.3	
Full‐time carer	7	13.0	
Not working	9	16.7	
In education	1	1.8	
Occupation			
Professional	9	16.7	
Semi‐skilled	12	22.2	
Unskilled	17	31.5	
Full‐time carer/not working	16	29.6	
Education – (those with degrees)	15	27.8	
Children (*n* = 41)^c^			
Female	17	41.5	
Age – all children			8.4 ± 2.5, range 2–12
Female age at time of interview (years)			9.0 ± 2.2, range 5–12
Male age at time of interview (years)			8.0 ± 2.7, range 2–12
Female age at diagnosis (years)			5.2 ± 2.1, range 3–10
Male age at diagnosis (years)			3.6 ± 2.3, range 1–8
Diabetes duration – all children (years since diagnosis)			4.1 ± 2.9, range 1–11
Regimen (at time of interview)			
Basal Bolus	26	63.4	
Mixed use insulin	2	4.9	
CSII	13	31.7	
HbA1c – all children (IFCC: mmol/mol; NGSP: %)			68 ± 12.3; 8.4 ± 1.1

a A total of 40 interviews were conducted. Of these, 24 interviews were with mothers only, 2 with fathers only and 14 were joint interviews with both mothers and fathers.

b Two parents, both mothers, had T1D.

c Details of 41 children are provided as one set of parents cared for two children with type 1 diabetes.

### Data collection

A total of 40 interviews were performed, including 26 solo interviews (24 with mothers and two with fathers) and 14 joint interviews (involving both mothers and fathers). Interviews took place between November 2012 and August 2013 and were undertaken face‐to‐face in parents' homes. All data were collected by DR, who has extensive experience of conducting interviews involving sensitive content and knowledge of T1D. Interviews were informed by topic guides developed in light of literature reviews, original research questions and inputs from members of the study's advisory group, which included health professionals, policy makers and parents of children with T1D. Topics explored in the interviews are shown in Table 2. Interviews averaged 120 minutes, were digitally recorded (with consent) and transcribed in full.

**Table 2:** Aspects of support explored using the topic guide

Setting the context. Parents were asked about: Accounts of their family‐ and work‐life History of diabetes in the family Accounts of, and reactions to their child's diagnosis
Questions about support. Parents were asked about: Experiences and views about the information, support and advice that they had received from health professionals when their child was diagnosed; views about when this support was provided; what they had found helpful and unhelpful. Experiences and views about education or training that they had received from health professionals around diagnosis to help them to manage their child's diabetes at home; what they had found helpful and unhelpful. What were their own needs for support at the time of diagnosis and whether/how were these addressed; what were their unmet needs for support Examples of additional support or education they would have found beneficial around the time of diagnosis. Experiences and views about diabetes‐related support they had received when their child was discharged from hospital; when was support sought/provided; what they had found helpful and unhelpful; what were their unmet needs. Whether and for what reasons they had sought any other forms of support aside from the help provided by health professionals. Examples, and experiences, of seeking and receiving alternative forms of support.

### Data analysis

A thematic analysis was undertaken by two experienced qualitative researchers (DR and JL) who performed independent analyses, reading each participant's interview in full. Participants' accounts were also cross‐compared using the constant comparative method^29^ to identify issues and experiences which cut across different parents' accounts. Joint meetings were held after independent analyses had been undertaken to compare interpretations, explore parents' underlying reasoning, resolve any differences in interpretation and reach agreement on recurrent themes and findings.^29^ These meetings were also used to develop a series of codes which reflected the topics explored with participants and emergent themes. NVivo, a qualitative software package (QSR International, Doncaster, Australia), was used to code and retrieve data. Coded datasets were printed out to be read and subjected for further analyses to identify further themes and subthemes and illustrative quotations. Below, data are tagged using unique identifiers with the letter ‘M’ or ‘F’ signifying a child's mother or father respectively.

Ethical approval was provided by the South East Scotland Research Ethics Committee 01, NHS Lothian (12/SS/0071).

## Findings

Parents reported similar experiences of diagnosis, emotional impacts on themselves and accounts of how they could be better supported, irrespective of the length of time which had elapsed since their child had been diagnosed. Most began by describing how their child had been diagnosed with T1D by the family doctor and then admitted to and treated in hospital for between 2 and 8 days. During this time, parents described how their child's blood glucose levels were stabilized and they were given instruction and education on how to manage their child's T1D at home. Parents were also provided with contact details of the diabetes team and out of office hours telephone numbers if they required advice about managing their child's diabetes at home. Below, we explore parents' accounts of information and support received after their child was diagnosed, the challenges they encountered when they first began to manage diabetes at home, their unmet needs for support, and their suggestions for how other parents could be better supported in future.

### Experiences of information provided during hospital admission

#### Information overload

As we have reported elsewhere,^30^ many parents described being very distressed when they were informed about their child's diagnosis, particularly when this was accompanied by life‐threatening diabetic ketoacidosis. In the initial days after admission, all parents praised their child's clinical care and the detailed instructions given by staff on how to perform injections, monitor blood glucose levels, detect signs of hypo‐ and hyperglycaemia; and, in some instances, count carbohydrate in food. However, with the exception of those who had T1D or were health professionals themselves, or who were well acquainted with other people who had the condition, parents highlighted the challenges and difficulties they had encountered understanding and assimilating information delivered using unfamiliar terminology:

he started talking about ketones and it was like, ‘what, huh?, pardon?’ Total foreign language (013M);

I got very upset because we used to get taken each day into a room and be given all this training… I felt like I was an Arts student who had been thrown into a medical lecture theatre (017M).

As well as struggling to understand clinical terminology, parents frequently described feeling overwhelmed by health professionals' instructions and advice: “it was such a blur because it was all so much information at once” (001M). Many parents also discussed how they had felt stressed and extremely upset after finding out their child had T1D, and how, as a consequence, they had been unable to assimilate and retain any of the information and advice imparted to them in the hospital:

they gave me leaflets to read while we were waiting but, it was, I was in shock and that, I wasn't absorbing anything I was reading or listening to (022M);

she was only three, she was screaming the place down… and, to be honest, it was in one ear and out the other (034M).

#### Parents' need for more reassurance and emotional support after diagnosis

Despite feeling overwhelmed and, hence, struggling to absorb information, virtually all parents recognized their need for regimen‐specific information before their child could be discharged: “you feel devastated but you've got to get over that and, you're like ‘right, what have I got to do?’” (004M); “it's a massive learning bit and you have to learn instantly, there's no gradualness, really” (002M). However, because of their state of shock, many parents described how it would have been better if health professionals had given them reassurance and emotional support in the first instance:

it was so much practical information, you know, this is how you keep him alive, this is about carbohydrate. It was like, but actually I wanted to know, is he going to die? … I really know that it is serious but I think, for a parent, it would have been good to have a little bit of the comforting, like ‘you're going to be okay’ (006M).

Other parents, likewise, reported needing emotional support to address their initial concerns and anxieties following their child's diagnosis and admission to hospital and described how reassurance given upfront might have made them more receptive and better able to assimilate practical advice thereafter:

just in layman's terms, this is what's wrong, this is how we're going to treat her, she will get better as in she's not going to die and then… when everything's calmed down and you, your relief's sort of like swept over you … then start explaining, right, you have to start to give her injections, the BG [blood glucose], the meters, then start going through all the equipment. (002F).

### Returning home with a child newly diagnosed with T1D

Many parents described how health professionals' advice had left them unprepared for how having a child diagnosed with T1D was likely to affect their lives with several suggesting that they would have benefited from being given information in this regard:

when it was first diagnosed… you don't want to just hear about the medical side, you want to hear more, you want to see how this is really going to impact your life (023M).

Indeed, the challenges involved in managing a child with T1D often only became fully apparent when parents first returned home with their child, a situation which 003M, like others, likened to one where:

somebody gives you a massive book for a computer and says, you know, ‘you've now got to start work with it straightaway’.

#### Concerns about administering injections

Parents reported several key challenges soon after diagnosis, including having to convey information about diabetes to young children and explain the need for daily injections: “how do you tell a five year old he's going to have to be injected four times a day?” (010M). Whilst all parents recognized that their child's life depended on them administering insulin, many described traumatic and distressing experiences where they had had to chase after and physically restrain a child who resisted injections:

I would have to literally pin him down; I would have his legs between my knees to, to be able to do it (015M);

he would smash up the house ‘cause he didn't want his injection… and I do remember quite often just sobbing, just sobbing in the corner …thinking, ‘oh God, nobody knows what this is like.’ (006M).

These parents often described initially dreading injections because of their child's resistance to being injected whilst those with larger families expressed concerns about how siblings might be affected: “I don't think it was good for them to see me having to pin down their big brother and see him screaming” (016M). They also spoke about needing, yet struggling, to obtain information and support on how to handle these potentially distressing situations and to pre‐empt and prevent their child's fear and upset.

As well as having to deal with their child's distress, some parents reported suffering from needle phobia themselves or feeling so concerned about inflicting pain on their child that they struggled to perform injections. In some instances, this led to parents going to extraordinary lengths to psychologically prepare themselves before they were able to administer an injection and to their feeling isolated and unsupported:

if I don't do this right, I could end up doing something to her … and it used to take me an hour to set up needles in the kitchen just to get myself psyched up to come in and jag her… I was just kind of stalling it… I could have done with someone coming round to help (037M).

#### Concerns about nocturnal hypoglycaemia

The majority of parents also reported feeling very concerned that their child would not detect symptoms of hypoglycaemia when asleep, fail to wake up and die in bed. Whilst most felt able to monitor their newly diagnosed child for signs of hypoglycaemia during the day, many described only sleeping lightly and, as Sullivan‐Bolyai *et al*.^26^ has also reported, remaining in a constant state of alert at night: “I slept in beside him for the first few weeks, just sort of monitoring him and I wasn't really sleeping” (005M). Such parents described feeling exhausted as a consequence:

when we were up very much every night one, you feel sorry for [daughter] … and two, you feel sorry for ourselves because we're not getting any sleep (017F).

Others also described how their sleep had been disrupted at regular intervals because they had set alarms to check on their child or because they had been too frightened to go to sleep at all: “I just couldn't sleep because I just, well, I wanted to see, was checking her all the time” (034M). In addition, several parents described how these concerns had even pervaded into the following day when they went to wake up their child:

I would test her, I would go through the motions at night, really, to be there… and sometimes I would go through in the morning, try and wake her and if she was in quite a deep sleep, cause she can be in a deep sleep, I was, my, your heart pounded, you know, and your stomach… (037M).

### Unmet needs for support when caring for a child at home

#### Emotional support

Although most parents praised the extent of the telephone support provided by health professionals to help them manage their child's diabetes at home (e.g. to adjust insulin doses; advice on treating hypoglycaemia), several reported that:

there was absolutely nowhere to go, nobody to turn to, there was nobody to speak to you about how you were actually feeling about the situation (032F).

Whilst a few parents described seeking and receiving emotional support from members of their child's diabetes team during clinic appointments, others described being reluctant to ask for help because “you don't really have a relationship with the people you're dealing with” (008M) or, more typically, because they were worried that they would be perceived by staff in negative ways. This included 006M who did not share her feelings of anguish and depression with health professionals when her son was diagnosed because of her concerns she might be seen as a failed parent:

you need someone just so that you can sit there and be open and it not be, feel like you're being judged, cause if you say it to a nurse or a diabetes doctor, the last thing I wanted was for them to think well, [son's name] mum is not coping and so, therefore, that raises alarms… I wanted him to be at home. I wanted life to get on.

Whilst most parents praised the accessibility of their child's diabetes team by telephone, many also described how they would have preferred it if clinicians had initiated contact with them, particularly in the early days after returning home:

I think that at that point you need it thrust upon you, they need to be in touch with you, every day, they need to actually speak to you rather than you having to phone them, they need to phone you and say, ‘look, how is it going?’ Just so you know there is somebody there (002M).

This included parents who suggested that they would have benefited from home visits from health professionals in order to observe how they were implementing their child's insulin regimen:

I wonder sometimes if there was … somebody who came out just to see how things were going that weren't exactly about his [blood glucose] numbers… I can massively see how you would just think, ‘ah, to hell with it’ …or ‘och, if it's high, we'll just give him insulin later’ you know, and just not bother (025M).

Others described how health professionals could use home visits to ascertain how they were coping and if they needed to be offered emotional support. This included 021M who reported feeling depressed after her child was diagnosed and who suggested that she would have benefited from home visits modelled on the service provided by midwives to new mothers:

to make sure the baby's alright and that you're alright as a parent” but with a broader remit than the child's glycaemic control: “just someone to come round to say, ‘what has been happening?’ so that you know there's someone there not just about blood levels … but it was also having more an impact on [my husband] and I because we were seeing it, I suppose, like death for a child.

#### Practical support for parents

In addition to emotional support, parents also talked about needing practical help delivered in the home setting, which took into account their family circumstances and the challenges of implementing their child's regimen in ‘real life’ circumstances. For instance, several parents who had received instruction in carbohydrate counting when their child was in hospital described how they would have benefited from home‐based support with an emphasis placed on exploring what the family ate: “I would literally take them [health professionals] into my kitchen and say, this is what's in my cupboards” (0021M). Others, as described above, highlighted a need for advice about how to inject their child without causing them upset and distress:

it was six months before I actually felt really confident [to inject] but, you know, I'm sitting saying to [partner's name] somebody should maybe be out, like a consultant to the house… Not one person's come out to the house to see that we've done it all the way it should be done. (001M).

#### Peer support from other parents with a child with T1D

Whilst all parents spoke about needing and/or valuing clinical input when they first returned home with their child, several suggested that health professionals were not ideally placed to provide the empathetic and non‐judgemental forms of support they saw themselves as requiring, because they lacked real‐life experience of caring for a child with T1D:

although they're a medical professional or whatever, yes they have, you know, empathy, etc. … but they still can't appreciate what it's like (014M).

Hence, such parents suggested that they would have benefited, soon after returning home, from speaking to:

somebody that really understands how diabetes eats into your world… I don't think I even needed support to manage it, I think I needed someone to come and give me a big cuddle, really, more than anything else because so much of it is about coping and making life good for [son] (013M).

Some parents also described how they would have benefited from opportunities, soon after diagnosis, to meet and talk with other parents who had experience of caring for a child with T1D. Others, likewise, suggested that experienced parents could provide those whose child had been newly diagnosed with practical suggestions and advice about the day‐to‐day issues involved in managing diabetes and how to deal with novel situations such going on holiday. This included 015M who described how peer support could be useful in situations where advice from a health professional was not required:

just someone else to speak to if you've got a problem, not necessarily a medical problem but, say something, you know, you're struggling to get your child to eat … what do they do when they come up against these things (015M).

Furthermore, parents suggested that those with recently diagnosed children might benefit from emotional support provided by peers who had experience of caring for a child with T1D and who could offer reassurance: “that the emotions that, that you'll go through, and the tests to your relationship, are all pretty normal and you will get through it” (011M). In addition, parents described how they might benefit from opportunities to talk openly to experienced parents without worrying about being seen as a failure by their child's clinicians: “you can sort of, offload a lot more stuff to somebody else that's in your position” (010M).

Other parents, however, described having been very upset and traumatized by their child's diagnosis and needing time alone to make adjustments which led some to question whether they would have been receptive to peer support because: “at diagnosis, everything's that sort of crazy… the thought of going to talk about it might have even been a bit much” (016M). In some instances, parents described having received and declined further opportunities to access peer support after meeting other parents at organized events for children with diabetes, or after being introduced to peer parents (e.g. by health professionals), and having encountered people who they described as obsessed and overbearing or whose advice could be contrary to that provided by clinicians. This included 007M who described an unhelpful meeting with another parent where she had disagreed with the peer parent's restrictive approach to her child's diet:

we'd ordered a healthy food platter but she was going, ‘no, you can't have that grape’… and I was sitting there thinking ‘Oh my God, that kid's going to grow up with food issues’ … and I thought, that's just making me anxious, it's not giving me the support that, that I was looking for so I gave up on it

## Discussion

This study explored in‐depth parents' views about the information, advice and support received after their child is diagnosed with T1D and is one of the first to explore their accounts of the timing and chronology of current support provision, and how this support could be improved in order to better care for their newly diagnosed child. Many of the parents who took part in our study reported feeling overwhelmed when their child was diagnosed and needing more emotional support prior to receiving practical instruction and regimen‐specific advice to manage their child's diabetes. Parents also described struggling to implement clinical and dietetic aspects of their child's regimen when they first returned home and how they would have benefited from more practical and emotional support from health professionals or from other parents, who, as some of those interviewed suggested, might be better placed to offer empathetic support.

Whilst parents' emotional reactions to their child's diagnosis, and the challenges they confront attempting to assimilate information, have been extensively documented,^12^, ^13^, ^16^, ^19^, ^22^, ^31^ our findings suggest that parents would benefit from having specific worries and concerns addressed in the first instance, such as their fear that their child might die, before receiving education about diabetes management. Specifically, our findings highlight the importance of health professionals spending time with parents soon after diagnosis, asking about their existing knowledge and understanding of T1D using lay and easy to understand terminology, and, when appropriate, providing them with reassurance about their child's condition, in order to better prepare them to assimilate complex regimen‐specific information to enable them to manage their child's diabetes effectively at home.

Our findings, in line with those from other studies, also highlight parents' need for more emotional support and practical advice in order to help them to adjust to and integrate their child's new regimen within everyday family life.^14^, ^16^, ^17^, ^18^, ^19^, ^31^ Unlike these other studies, however, we have shown that parents may not ask for, or access, the help they need. Specifically, we have shown that parents may feel reluctant to approach health professionals and admit that they are feeling unable to cope, or that they question whether health professionals, given their lack of personal experience of parenting a child with T1D, can provide the empathetic and non‐judgemental support which some of those interviewed described themselves as needing. To address the former, health professionals could consider offering proactive support in the first weeks following diagnosis; for example, through scheduling home visits and/or initiating phone calls with parents. To address the latter, the provision of peer support interventions, delivered by experienced or “veteran” parents,^32^ may be a potentially fruitful avenue to pursue. Indeed, the need for this kind of support was highlighted by some of the parents we interviewed. However, evaluations of peer support interventions for parents with children who have T1D,^33^, ^34^ and a range of other chronic conditions,^35^, ^36^, ^37^, ^38^ have thus far shown mixed results. Whilst parents have generally articulated positive views about receiving peer support,^39^, ^40^ researchers have been unable to demonstrate conclusive positive effects using psychosocial and other quantitative measures, such as parental stress and diabetes‐related concerns.^34^, ^41^ Whilst it has been argued that better measures may need to be developed to capture and quantify the true benefits of peer support for parents,^34^, ^40^, ^42^ our findings suggest that a possible reason for the lack of positive effects may be because peer support does not suit all parents. Not only may parents be put off by negative experiences, they may also be exposed to information which contradicts clinical recommendations. Hence, an alternative option to explore might be to provide health professionals with experiential training in caring for a child with T1D that would enable them to offer more empathetic and individualized support to parents. Whilst patients' adherence to medical regimens is known to be affected by doctors’ empathic communication skills,^43^, ^44^ there has been no research to date undertaken to explore the impact of experiential skills‐training in caring for a child with T1D for health professionals who provide consultations to parents of these children; hence this may be an important avenue to pursue.

## Strengths and limitations

The study was strengthened by the use of a multi‐site recruitment strategy, involving parents from four clinics serving diverse geographic areas, which improves the generalizability of our findings. A potential limitation is the inclusion of parents of children diagnosed up to 11 years previously which could mean that some were not describing contemporary practices and/or their accounts were subjected to recall bias. This potential problem could be addressed in future studies by including parents of children who have been recently diagnosed. However, it should be noted that the parents who took part in our study presented similar accounts and described similar support needs irrespective of the length of time since their child's diagnosis. A further limitation is that in our study all children diagnosed with T1D were admitted to hospital whereas, in some areas, children who are not diagnosed in DKA may be managed without admission to hospital.^45^, ^46^ Hence, future work could explore and compare parents' experiences when initial care and education is provided in outpatient settings or in the home.

## Conclusion

Our findings have important implications for service development and indicate that parents of children diagnosed with T1D might benefit from a package of support which extends from when their child is first diagnosed and admitted to hospital through to the weeks and months after they return home and begin to integrate their child's regimen into everyday family life. For instance, our findings highlight a need for more attention to be given to the timing and chronology of support offered to parents, in particular for parents to be offered emotional support soon after diagnosis to better enable them to assimilate diabetes management information at a time of great distress. Health professionals should also consider ways to provide more practical support to parents soon after they return home with their child, to help them integrate diabetes management into their family's normal lifestyle.

## Conflict of interest

The authors have no conflict of interests to declare.
